# Manipulating ovarian aging: A new frontier in fertility preservation

**DOI:** 10.18632/aging.100269

**Published:** 2011-01-24

**Authors:** Kutluk Oktay, Sumanta Goswami, Zbigniew Darzynkiewicz

**Affiliations:** ^1^ New York Medical College, Valhalla, NY 10595, USA; ^2^ Albert Einstein College of Medicine of Yeshiva University, Bronx, NY 10461, USA

Both the premature and age-induced ovarian failure are associated with significant medical and quality of life complications. Moreover, millions of children and adults suffer from iatrogenically-induced premature gonadal failure as a result of chemotherapy treatments. In the case of chemotherapy, commonly used cancer drugs such as doxorubicin or cyclophosphamide result in apoptotic death of primordial follicle reserve [1-3], probably by inducing double strand DNA breaks concurrent with intense activation of ATM-mediated DNA repair pathways (unpublished data). Clinically, females who receive similar cancer drugs and who do not immediately become menopausal will experience premature menopause because of the diminishment of ovarian reserve. The prevailing dogma dictates that the primordial follicle reserve is fixed *in utero* and any insult reducing such reserve causes irreversible harm as oocyte regeneration is not possible in postnatal life. This dogma has been seriously challenged by several independent laboratories in recent years [4-5], even though some have expressed difference in opinion.

While there has not been direct laboratory evidence in humans supporting or refuting the concept of germ cell renewal, our clinical and laboratory observations lend support to the existence of oocyte regeneration in human ovaries [6-7]. Ovarian cryopreservation is an experimental fertility preservation strategy which allows conservation of primordial follicle-rich ovarian tissue collected prior to gonadotoxic chemotherapy. After the completion of the cancer treatment and when fertility is desired, these tissues are thawed and transplanted back, enabling reversal of menopause. We have developed and performed first series of ovarian transplants in humans [8]. One of the ovarian transplantation techniques is transplantation of ovarian cortical strips under the skin [9]. In a cancer patient who became menopausal as a result of intense chemotherapy, we observed that transplantation of previously frozen ovarian cortical strips under the abdominal skin resulted in immediate reversal of menopause and four consecutive pregnancies with three live births [7]. This appeared to be the result of the activation of the remaining menopausal ovary in response to the transplantation of undamaged ovary under the skin. We speculated that chemotherapy is likely to damage the ovarian germ cell niche, as our earlier work suggested [2], resulting in the inability of the ovary to regenerate new oocytes. We surmised that the transplanted ovary provides the necessary regenerative signals from its niche to the preexisting ovary through the circulation, enabling this chemotherapy- damaged ovary to reinitiate oocyte generation (Figure 1).

**Figure 1.**
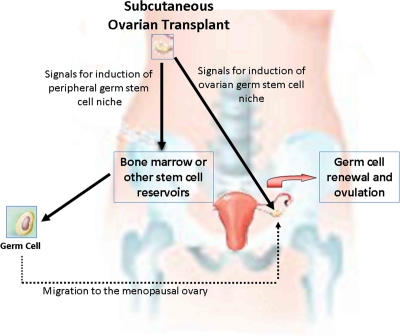
Hypothesis for spontaneous pregnancies after subcutaneous transplantation of ovarian tissues. Based on the work cited in this manuscript, we hypothesize that the transplanted healthy ovary provides the missing regenerative signals to peripheral niches and/or to the stromal niche in the remaining menopausal ovary that result in the resumption of neo-oogenesis.

Our clinical observations are in line with the parabiotic model reported by Niikura*et al* [10], in this journal and with their earlier work on the role of ovarian niche in oocyte regeneration indicating that external factors in circulation can affect ovarian niche to reinstate oocyte production in the aging ovary. It is interesting that the regenerative signal originates from the aged male mice. One wonders if androgens have any role in the oocyte regenerative effect that Niikura *et al* observed. Perhaps higher androgen levels in the circulation from younger males are responsible for increased follicular atresia that the authors observed, as this is a known effect of potent androgens. As the androgen levels drop in the senescing mice, regenerative effects of androgens on germ cells become predominant, resulting in overall increase in primordial follicle reserve. This hypothesis is also consistent with recent clinical observations that weak androgens may be associated with improved ovarian follicle reserve. DHEAS, a weak androgen, is associated with improved ovarian response to fertility drugs and enhanced ovarian reserve in aging women [11], and Polycystic Ovarian Syndrome, a disease of ovulatory dysfunction and mildly increased androgens, is assoc-iated with increased ovarian reserve [12].

As such the recent work by the Tilly laboratory [13] and our ongoing translational work open up a new frontier in the field of fertility preservation: manipulation of ovarian aging. The next era in fertility preservation research will be focusing on the pharmacological and genetic modifications in both slowing down oocyte attrition and enabling a healthier ovarian niche. This will carry the aim to not only enrich the primordial follicle pool but also reverse age-induced alterations in oocyte quality and possibly curb age-related infertility and pregnancy losses.

## References

[R1] Oktem O, Oktay K (2007). A novel ovarian xenografting model to characterize the impact of chemotherapy agents on human primordial follicle reserve. Cancer Res.

[R2] Oktem O, Oktay K (2007). Quantitative assessment of the impact of chemotherapy on ovarian follicle reserve and stromal function. Cancer.

[R3] Soleimani R, Heytens E, Oktay K (2010). Prevention of chemotherapy-induced apoptotic follicular death in human ovary by ceramide-induced death pathway inhibitors. Fertil Steril.

[R4] Johnson J, Bagley J, Skaznik-Wikiel M, Lee H-J, Adams GB, Niikura Y, Tschudy KS, Tilly JC, Cortes ML, Forkert R (2005). Oocyte generation in adult mammalian ovaries by putative germ cells in bone marrow and peripheral blood. Cell.

[R5] Zou K, Yuan Z, Yang Z, Luo H, Sun K, Zhou L, Xiang J, Shi L, Yu Q, Zhang Y, Hou R, Wu J (2009). Production of offspring from a germline stem cell line derived from neonatal ovaries. Nat Cell Biol..

[R6] Oktay K, Oktem O (2007). Regeneration of oocytes after chemotherapy: connecting the evidence from mouse to human. J Clin Oncol..

[R7] Oktay K, Turkcuoglu I, Rodriguez-Wallberg KA (2010). Four spontaneous pregnancies and three live births following subcutaneous transplantation of frozen banked ovarian tissue: What is the explanation?. Fertil Steril.

[R8] Oktay K, Karlikaya G (2000). Ovarian function after transplan-tation of frozen, banked autologous ovarian tissue. N Engl J Med..

[R9] Oktay K, Buyuk E, Veeck L, Zaninovic N, Xu K, Takeuchi T, Opsahl M, Rosenwaks Z (2004). Embryo development after heterotopic transplantation of cryopreserved ovarian tissue. Lancet.

[R10] Niikura Y, Niikura T, Tilly JL (2009). Aged mouse ovaries possess rare premeiotic germ cells that can generate oocytes following transplantation into a young host environment. Aging (Albany NY).

[R11] Sonmezer M, Cil AP, Oktay K (2009). Ongoing pregnancies from early retrieval of prematurely developing antral follicles after DHEA supplementation. Reprod Biomed Online..

[R12] Nikolaou D, Gilling-Smith C (2004). Early ovarian ageing: are women with polycystic ovaries protected?. Hum Reprod.

[R13] Niikura Y, Niikura T, Wang N, Satirapod C, Tilly JL (2010). Systemic signals in aged males exert potent rejuvenating effects on the ovarian follicle reserve in mammalian females. Aging.

